# Exploring the ethics of political PR professionals using moral foundations theory

**DOI:** 10.1371/journal.pone.0286217

**Published:** 2023-06-12

**Authors:** Oxana Mikhaylova, Roman Abramov

**Affiliations:** 1 Faculty of Social Sciences, HSE University, Moscow, Russian Federation; 2 Russian Academy of Sciences, Moscow, Russian Federation; 3 International Laboratory for Social Integration Research, Moscow, Russian Federation; Xiangtan University, CHINA

## Abstract

This paper offers a multidimensional theoretical scheme to analyze professional ethics in the field of political public relations. We suggest investigating the decisions of these professionals using moral foundations theory because human ethical reasoning is contextual, and the examination of ethics in a one-dimensional manner as previous researchers have done overlooks the complexity of the moral choices that such professionals make. The prospects of the proposed theoretical approach are demonstrated on 16 interviews with post-soviet Russian political PR industry leaders that were conducted from March 2018 to April 2020. Our empirical findings show that Russian political PR specialists employ all moral foundations, however, in their narratives the “care/harm” and “authority/respect” foundations were not mentioned very often. Overall, this paper makes a critical contribution to research on professional ethics in political public relations, and it provides important insight into the specifics of moral reasoning in the Russian political PR industry that is insufficiently described in the current literature.

## Introduction

The moral reasoning of public relations (PR) professionals is a significant focus of research for practitioners and researchers worldwide [1–3]. Although many studies describe the ethics of PR specialists in various fields of practice, the literature that addresses the ethics of political PR professionals in non-western cultural contexts is scarce [4–7]. Research on the professional ethics of PR specialists is based mostly on universalist or one-dimensional models of ethics inherited from the theoretical views of morality developed by Kohlberg and his followers [8–11]. Such an approach to measuring professional ethics among political PR specialists underestimates the contextual character of human ethical choices.

Our paper addresses these two problems. In particular, we use the scheme for moral foundations developed by Haidt and his colleagues [12] to investigate the ethical reasoning of political PR specialists in post-soviet Russia. The empirical base comprises 16 semi-structured interviews conducted from March 2018 to April 2020 among leading Russian independent political PR consultants, owners of firms, and self-described “spin doctors”. Consequently, the study contributes to the literature on political PR professionals’ ethics because we show that it has a multidimensional nature and that it is critically important to investigate professional ethics of these people contextually. Also, we add to the descriptions of the post-soviet political PR profession.

The article starts with a historical overview of the formation and transformation of the Russian political PR industry. It continues with an analysis of the literature on the ethics of political PR professionals. After that, the current study is introduced. Then the results are presented and discussed, and directions for future research are proposed.

## The political PR industry in post-soviet Russia: Historical transformation and the current state of affairs

The contemporary Russian PR industry began as a professional field and a market segment during the political and economic transition from socialism to capitalism in Russia and other socialist countries in 1980–1990 [13–16]. Its first steps of professionalization happened for two reasons: first, the emergence of demand for PR services among new Russian capitalists and politicians [7, 17–19] and second, actions taken by academic elites that managed the Russian university (MGIMO) who focused on training international relations specialists [7, 19–21].

*During 1990–2000* different actors in the PR industry tried to formalize ethical principles in the form of codes, declarations, and agreements [19]. In addition to this, in 1995–1996 political elections for the president and parliament took place [22, 23]. On the one hand, these elections helped to professionalize the PR industry in Russia [24, 25]. Apart from getting “real” experience working in the field, preparing for elections stimulated a valuable exchange of knowledge and practice between foreign and Russian PR specialists. On the other hand, these elections were famous for the extensive use of the so-called black PR technologies. Black PR technologies are unethical and cynical means used to achieve high revenues and financial prosperity (for instance, disseminations of lies and gossip about opposition political candidates, publishing distorted data of public polls in the media and illegal cancellation of agitational events). In the basis of these technologies lie media and psychological theories of consciousness manipulation and propaganda. Their use was widespread in the Russian political PR industry of the 1990s. Their extensive employment in professional practice was possible because of the low level of legal regulation of the Russian PR industry at that time. Now, the professional enterprise of political PR in Russia is much more legally regulated and the professionals mainly refer to the term “black PR” to compare the transparency and official character of their current practice with earlier difficult times in the history of the profession, when the political PR practice was functioning in anomical political and social contexts [5]. Therefore, it is supposed that these elections contributed to the formation of the public image of PR specialists as opportunist, corrupt, cynical, and adventurous professionals. In particular, some experts think these elections opened the space for the media manipulation and administrative pressure on Russian voters, hindering the development of democratic rights [26–30].

*During the early 2000s*, the Russian political PR segment finally established itself as a professional community and market segment, despite the economic collapse and economic crisis of 1998, and agencies in the capital cities (Moscow and Saint-Petersburg) continued to dominate the market [4]. In addition to this, large and medium-range businesses started to restructure, and the first in-house PR specialists appeared. They helped to communicate the images of the companies in Western markets. Since the 1990s, foreign companies have come to the Russian markets and brought their cultural attributes. Because of this, English words such as “image,” “publicity,” and “stakeholder” proliferated among Russian professionals [31]. These words became indicators of inclusion in the professional community, and they created an appealing and mysterious image of the profession among laypeople. Such perceptions of PR professionals were reflected in many well-known Russian books of fiction, like *Generation P* [32]. Despite the westernization of the professional community, it kept certain local traits [31]. First, it was still fragmented, and the influence of professional associations was minimal; these associations functioned mostly as public image creators. Second, political consulting and political PR became even more deeply intertwined with government, and many famous PR specialists came to work in government structures. Third, the PR market in Russia was developing under the same rules as many businesses, and it was typical for business structures to be in a gray zone and be engaged in illicit practices [33]. This historical period is still scarcely treated in academic literature.

*During 2010–2020*, in the Russian PR industry, there were two controversial processes. On the one hand, there was a massive extension of governmental involvement in the economy, including the political PR sector, and a massive restriction of political freedoms [34]. Therefore, large political PR agencies started to target their services to the governing party “United Russia” or to state functionaries. On the other hand, that decade was revolutionary because of the transformation of mass media, also called the technological revolution [35–37]. Both international and local Russian mass media became important for Russian social life [38]. Many of these processes echo the “global village” and “global theater” concepts of [39], when everybody could be a producer of media content that will be popular among millions of people [40]. In these circumstances, old theories and technologies of influence worked only partly, so the content of professionals changed and the younger echelons worked with these new technologies [41–44]. Gradually, government agencies became more advanced in their techniques of political manipulation on the Internet, not only in Russia but also in the global context. For example, professionals and the lay public actively disputed the role of Russian people in the elections of 2016 and 2000 in the USA and whether they were favorable to Trump [45–48]. These suspicions significantly deteriorated relations between the USA and Russia. Overall, governmental invasion in Russian politics went hand in hand with the technological revolution and created new ethical challenges in this field.

Generally speaking, contemporary Russian political consulting and PR have developed a fully functional institutional infrastructure [49]. It includes professional associations, the system of education, and documents that regulate the professionals’ codes of ethical conduct. At the same time, the professional community of political PR specialists in Russia is weak, and interactions within factions are based on friend and partner networks. Also, it could be argued that the formal codes of ethics in Russian political PR mostly serve as the image component of the profession. In practice, the practitioners follow informal perceptions of what is right and wrong and traditions of business and professional communication culturally formed in Russia.

### Political public relations ethics in the scope of academic interest

The ethics of PR specialists is one of the most important topics to be investigated by social science and media scholars and practitioners because moral norms determine the internal integrity of this profession and the public trust in services and products it presents to them [1–3]. Even though we know a lot about PR professionals in general, less is understood about the ethical aspects of PR practice in politics. The professional activity of political consultants, advertisers, and marketers can affect the political future of a country and its relations with other nations. Therefore, the formal regulation of their decision-making with ethical codes and other legal documents is especially important for the public good and promoting and maintaining democratic values. Political PR specialists, as well as other media professionals, behave in relation to the professional, personal, and environmental contexts [50, 51].

### Professional values and work-related contexts of ethical decision making

PR professionals must make various ethical choices at work, and these choices tend to change over time [52]. Neill, for instance, discovered 10 moral issues that people who work in this area must resolve. Some scholars state that telling lies (information distortion) is the inevitable aspect of political PR practice [53–55]. Moreover, it is claimed that the question of ethics codification arose among PR specialists only because of the need to receive more credit and support for their role in the eyes of the public [56, 57]. In turn, the practice of lying by political PR agents is multifaceted: these people have developed various tactics of lying [53].

Moreover, PR specialists are prone to be morally disengaged because their position in the chain of the political process is an intermediary one. Studies in various countries show that these specialists evaluate their accountability differently, based on the ethical character of their clients. Some practitioners suppose that it is up to the client to draw the line between moral and immoral political actions [58–60]. However, the ethical values of politicians and PR specialists differ at statistically significant levels [61] (Waller evaluated two dimensions: honesty and accountability). Also, in consulting firms, there is the problem of ethical individualization (it is not obvious who is held accountable for ethical dilemmas: the firm or the consultant). If the employees of the firm are responsible for the moral outcomes of consulting, this could lead to dangerous malpractice as too much pressure and relativism are imposed on individual consultants [62].

At the same time, some scholars treat PR specialists as supporters of ethical leadership, and they research the practice of PR specialists from that perspective [63]. They claim that PR professionals must evaluate their ethical decisions informally by “gut checking” [50]. They pose questions, think about the impact of their decisions on society, compare that with the values of the organization where they work, and think about the needs and possible reactions of the target public.

### Personal values and non-work-related contexts of ethical decision-making

Apart from their professional lives, many political PR specialists have other spheres, where they make ethical decisions (for instance, in romantic relationships and friendships). Measuring the moral choices that these practitioners make in non-professional contexts could reveal the tacit ethical principles on which they rely in work-related situations that are not regulated by professional ethical codes for various reasons. Moreover, the literature on PR professionals in general and on political PR per se shows that even when formal ethical codes are applicable these people tend to employ their moral compass instead of established ethical codes [51, 64–66].

The moral characteristics of PR specialists in non-work-related contexts were investigated mainly by moral development researchers. Moral development is the concept introduced and developed by Piaget, Kohlberg, Gilligan and their followers [8]. Their research is the basis of the Defining Issues Test (DIT) that these researchers of the moral development of professionals employ. Simply put, this test helps them understand the percentage of time people use universal principles in their reasoning. These studies showed that people who work in PR, irrespectively of gender, age, education, and party identification, usually score very high on moral development tests compared with the other professionals, and their moral development is comparable to medical professionals and journalists [8, 9, 11]. However, other research has shown that PR professionals, who are more liberal in their political and religious views, work in larger organizations, belong to a smaller number of professional organizations, value external ethical guidance less, know the professional ethical codes, and say that truth and respect are important to them. PR professionals are more idealistic than relativistic (in this research, the less idealistic behaved more unethically) [9]. In addition to this, older practitioners have been found to be less relativistic. Gender and education did not affect the degree of relativism of PR specialists.

### Environmental values

The moral decisions of PR political professionals could depend on organizational and cultural values. The cultural influence at the country level is visible, first, from comparisons to global ethical codes. For instance, [67] showed that Russian codes of ethics are centered on three values: expertise, advocacy, and relationship building. Other values such as professionalism, moral standards, and client’s rights are valued less. Also, the importance of cultural context is usually shown by the cultural dimensions comparisons [68]. As for the impact of the organizational ethical climate, there is a difference in the sources of ethical values that people employ in PR, depending on whether they are operating in the private or the public sector [69]. Those who work in the public sector rely more on corporate values and the public good. Conversely, in the private sector, PR professionals tend to be more focused on personal values, not on the organization’s ones. Moreover, PR specialists who work in larger organizations tend to behave more ethically [8]. The use and reliance on official codes are mixed because of resistance to be controlled, and the professional identity of this occupation is blurred [60].

Overall, from the research on the ethics of political PR professionals and PR professionals in general, it is apparent that the moral decision-making of these specialists has been extensively investigated in the separate areas of their lives, but the interrelation between those areas is not yet fully understood. Instead, we suggest using the moral foundations theory [70] to theorize and empirically research the moral attitudes of political PR professionals. Compared to the (unidimensional) moral development theory noted earlier, moral foundations theory presupposes that people’s ethics are multidimensional [12, 71, 72]. It posits that our moral attitudes are based on the foundations we activate in our decisions: fairness, care, authority, sanctity, freedom, and loyalty. All these foundations are bidirectional; they have negative and positive sides.

## Methods and materials

To discover the moral foundations of the political PR professionals, semi-structured qualitative interviews with these specialists were conducted from March 2018 to April 2020. The interviews were taken during the aforementioned time period because in 2018 almost 30 years passed since the beginning of PR industry professionalization in Russia [13–16] and some of this industry founders started to pass away or finished with the active work. Furthermore, in 2018–2020 the statues of limitations that political PR professional signed up in 1990-2000s expired and it began to possible to share the information about their past work. In addition to this, it is important to highlight, that the data harvest in 2021-till 2023 was constrained due to quarantine restrictions during COVID-19 epidemic. The informants were not willing to share their personal data in online interviews and face-to-face offline contact seemed to them dangerous as the risk of getting coronavirus was high, especially for middle-aged and older people, of whom our sample was comprised of. The participants were recruited using purposive typical case sampling [73].

As our study was focused on the ethics of post-soviet Russian political PR professionals that have worked in Russia in this field since its establishment, we searched for the leading Russian political PR market specialists that have been working in this market since 1990 and were engaged in PR specialists’ professional associations. Two sources of information about the market structure were triangulated: the Internet and books about leaders in the field. For the first, we contacted the most active members of the related professional associations. For the second, we examined books that provided overviews of key market figures like “Who’s who in PR?” [74].The aim here was to verify the backgrounds of the figures selected in the Internet site analysis. As a result, our sample includes 16 participants (14 male and 2 female), aged between 48 and 72 years old. Among those selected, there are owners of PR companies, independent political consultants, spin doctors, PR managers in companies, and a general director of the public opinion research center (Table 1).

**Table 1:** Demographic data on participants.

Participant	Age	Gender	Self-identified occupation
P1	65	M	PR company owner
P2	50	M	Independent political consultant
P3	47	M	Spin doctor
P4	53	M	PR manager
P5	60	M	PR company owner
P6	50	M	PR company owner
P7	72	M	Independent political consultant
P8	50	M	Spin doctor
P9	67	M	Independent political consultant who owns a PR company
P10	62	M	PR company owner
P11	59	F	PR company owner
P12	48	M	Former spin doctor, general director of the public opinion research center
P13	55	M	PR company owner
P14	50	M	PR company owner
P15	50	F	PR manager
P16	64	M	PR company owner

During the interview, informants were asked about their professional biography and the characteristics of the Russian political PR industry in 1990–2000 and its further transformation. In addition, they were questioned about professional ethics, the role of professional organizations, social media, and Internet technologies in this industry. At the end of the interview, they were asked their opinion of the current state of political PR and consulting in Russia. The interviews lasted between 35 and 90 minutes.

Interview data were analyzed using qualitative thematic analysis by two researchers [75]. We employed inductive semantic coding. One of the authors (author 1) read all transcripts and elicited the parts that related to ethical reasoning. After that, the other author (author 2) coded the fragments based on Haidt’s moral foundations (see the details on such analytical technique in [76]). Then the codes were revised by author 1, and the final thematic categories were defined in discussion with author 2. Some quotations have been modified slightly to improve clarity. The study was approved by HSE University IRB board, and all participants provided written informed consents.

## Moral foundations in political PR practice

The findings address five moral foundations. Three of them, “fairness/reciprocity,” “in-group/loyalty” and “purity/sanctity” were referenced more frequently during the interviews. The other foundations, “care/harm” and “authority/respect,” were mentioned less often. The themes and subthemes in the narratives are elaborated below.

### Theme: Care/harm

The participants expressed the importance of care/harm toward society, clients, and other political PR professionals, but they did not mention others that could be harmed/cared for because of their actions: individuals other than clients and non-humans. Examples and quotes in support of this theme and its subthemes are in Table 2.

**Table 2:** Examples of the care/harm theme and subtheme from interviews with political PR professionals.

Theme/subtheme	Participant	Example
*Care/harm toward other political PR professionals*	P16	“Historically, it said that you should work against the other candidate and not against his team. We did not humiliate each other.”
*Care/harm toward society*	P10	“I mean, not only do not humiliate each other but also have ethical principles toward the client and target audience.”
*Care/harm toward client*	P4	“There are three professions in the world in which the worse the client feels the better is for the professional. These are physicians, advocates, and PR specialists. . . I mean, that if you are ill, you could get addicted to pills. If you have certain problems, the more the advocate delays the process, the more he/she gets money per hour. The lengthier the political campaign is, the better it is for PR specialists.”

### Subtheme: Care/harm toward other political PR professionals

Four participants emphasized that in their practice they care about other PR professionals’ wellbeing. They said that they work against politicians, not against other PR specialists. This position of the interviewees could have pragmatic reasons behind it. As one participant stated, the PR specialists frequently work in teams with each other, therefore it is important to be polite to the colleagues.

### Subtheme: Care/harm toward society

Three participants claimed that they consider the public good in their professional decisions. It is important for these political PR specialists to consider how their actions will affect ordinary people, and these professionals bear in mind what is considered bad by the public. The interviewees also argued that professional ethics is grounded in universal assumptions about evil and good that are internalized during socialization. All the mentioned political PR specialists said that they try not to harm the public with their decisions.

### Subtheme: Care/harm toward the client

Two interviewees stated that they care about the effects of their deeds on their client’s state of affairs. One of them indicated that the worse the state of the client the more profit will come from handling the case. He also compared PR professionals to doctors and advocates because, to his mind, all these professionals could get more money for their services the worse the condition of the client is. Also, it was claimed that it is common that the borders between professional and personal relations blur, and therefore sometimes political PR professionals could care more about the state of their client when it is required formally. A second interviewee claimed that the good of the client is one of the goods that he tries to fulfill when he does his work, along with the public good and norms of behavior established between colleagues in his professional community.

### Theme: Fairness/reciprocity

Participants applied the notions of fairness and reciprocity to the same subjects about which they applied the concepts of care and harm (society, clients, colleagues). However, while talking about PR specialists, our interviewees tended to conceptualize their relations with colleagues as reciprocal. They mostly did not speak about honesty. Conversely, when they discussed the same topic concerning clients and society, they did speak about honesty/dishonesty. We think this relates to the backside logic that they use while thinking about these different types of relations. Namely, PR specialists are in economic relationships with them, clients have borderline relations and society is in non-professional relations with these people. (Some examples of subthemes are in Table 3).

**Table 3:** Examples of the fairness/reciprocity theme and subtheme from interviews with political PR professionals.

Theme/subtheme	Participant	Example
*Reciprocity in relations of political PR specialists*	P3	“If you get around somebody, people will remember that. . . We do not have a developed reputation market. However, everybody knows each other and is aware of coalitions and confronting parties. We have signaling lights. If you suddenly switch the side, market players will get even with you one day.”
*Honesty/dishonesty toward clients*	P8	“For instance, about financial and money matters in relations with clients, I always applied ethical maxims. It means I did not steal and rat on them.”
*Honesty/dishonesty toward society*	P4	“By deceiving public opinion or misleading people, you violate professional ethics. It means that you could be expelled from the PR professionals’ guild due to this formal ethical misconduct.”

### Subtheme: Reciprocity in relations of PR specialists

Eight participants endorsed the importance of the reciprocity in relations with PR specialists in the professional community. All of them argued that it is crucial to behave well toward the other PR specialists because it guarantees that you also will be dealt with well. Besides this, they said that behaving well contributes to their reputations in the field. The better you behave toward others, the higher your reputation.

### Subtheme: Honesty/dishonesty toward clients

Honesty with clients was addressed in the narratives of four people. By being honest with the client, interviewees meant being clear about their ethical principles, not distorting information, maintaining confidentiality, and being fair about financial compensation. One participant claimed that he intentionally does not sign up for ethical codes that presuppose honesty with clients because he thinks that is unprofitable in the current hostile Russian business climate. Another one mentioned that when she works with Russian political elites, signing such codes helps to gain credibility in their eyes because it is considered European and democratic.

### Subtheme: Honesty/dishonesty toward society

Two political PR professionals mentioned honesty with the public while speaking about professional ethics. By being honest, they meant not lying or distorting information. One person indicated that he is against lying to people, but another one claimed that he thinks it is normal to lie if it helps the client to win.

### Theme: In-group/loyalty

#### Subtheme: Loyalty to the political PR professional community

Participants mentioned that the most important group for them is their professional community, that is why the in-group/loyalty category referred mostly to this topic. We provide illustrations for this theme in Table 4. Twelve PR professionals mentioned that their ethical decisions are always based on the informal group norms of their professional community. All of them obey these norms. The norms of the field according to informants have formed naturally through time. Obedience to them helps to ensure their safety, all of them said, and two people claimed it helps to draw invisible borders between the players of the market that are us and that are them.

**Table 4:** Examples of the subtheme of loyalty to the political PR professional community from interviews with political PR professionals.

Participant	Example
P2	“If you will not order the murder of somebody, it is not guaranteed that somebody will not decide to kill you for a certain reason.”
P12	“In communication, we quickly understood that we are from the same cloth.”
P14	“When we created this agency, I mean, we could have had some arguments with our partners on some other topics, but as for the questions of ethics in business, and the ethics in our relationships with clients, mass media, we had a solid consensus, and the market considered us village idiots for trying to disseminate the idea of ethics and refuse to get additional money.”

### Theme: Authority/respect

The authority/respect foundation was mentioned rarely by our interviewees, and it related to their respect of political authorities, laws, hierarchy in client-professional relations, and hierarchy inside the political PR community. See Table 5 for examples.

**Table 5:** Examples of the authority/respect theme and subtheme from interviews with political PR professionals.

Theme/subtheme	Participant	Example
*Respect toward political authorities*	P3	“In political PR people from the government are always involved and these are either the president, his press secretary, or the head of presidential administration, and there are also regional governments with whom you also have to respect.”
*Respect toward formal governmental laws*	P16	“What is not prohibited by law is allowed. This means that something could be either legal or illegal. Black PR is a myth, hell knows what it is.”
*Respecting hierarchy in client relations*	P4	“Keeping the distance with clients and being in hierarchical relations means a lot for me. If you do not fraternize, you can prepare a fair bill. If you break this neutrality in relations, it will be morally difficult to argue: ‘Hey, add more money please!’ I had such an experience with blurred distance and was not satisfied with that. Usually, it is very difficult for people to be within the business limits.”
*Hierarchy inside PR community*	P2	“We signed up for certain papers, but this was more for the fixation of superiority for the community leaders. We wanted to show that all who are on the top need to sign up the papers, the others are nothing and with them, it is completely unnecessary to sign any papers.”

### Subtheme: Respect toward formal governmental laws

Five participants argued that they base their decisions on Russian law. All of them stated that they obey legal norms, and these norms primarily help them to orient between professionally ethical and unethical actions.

### Subtheme: Respect toward political authorities

Three PR professionals mentioned political authorities (government, president, municipal political figures) that must be respected when they make certain decisions. These participants claimed that they consider the authorities’ interests during their work.

### Subtheme: Respecting hierarchy in client relations

One person emphasized the hierarchy needed between him and clients in ethical professional relations because sometimes borders between professional and personal relations tend to blur.

### Subtheme: Hierarchy inside the PR community

Hierarchy exists in the political PR community, and it is indirectly visible through the need to sign ethical codes. This was mentioned by only one interviewee. This participant argued that those who signed codes want to show to the other market players that they are privileged and others who do not sign are “outside the system.”

### Theme: Purity/sanctity

The purity/sanctity foundation involved reflections about the existence or absence of black (dirty) PR compared to white (honest) PR. Almost no religious or spiritual language or religious connotations were used to conceptualize the boundary. Only one person cited the Ten Commandments to illustrate his point. Others tended to employ a narrow variety of words, mainly “dirt” or “black” when they addressed the topic (see Table 6).

**Table 6:** Examples of the black and white political PR subtheme from interviews with political PR professionals.

Participant	Example
P4	“I do not understand at all what is black/white PR. I always argued during my teaching at the university that PR could only be either effective or not.”
P5	“There was such a time when we made gifts and solved our problems using black PR. After that, we started to be more effective and understood that it is better to solve problems using the prosecutor’s office and investigation committee.”
P9	“All black technologies employed in PR are practically the reflection of the clients`moral principles.”

### Subtheme: Black and white political PR

Eight interviewees mentioned the notion of black or dirty PR and contrasted it with white. Three of them said that for them there is no black PR because the most important thing is what is effective and what is not and lying and distorting information is an inevitable part of their profession. Also, one participant claimed that there is no black or white PR because the ethics of PR reflects the clients’ values, not the PR specialist’s ones. Others argued that the indicators that define what is black PR are disobeying judicial codes and internal universal ethical principles. Also, two interviewees said that black PR is not in fashion now in Russia. Besides this, it was claimed that using black PR is has become risky for one’s reputation in the field. It could discredit you in the eyes of colleagues.

## Discussion and conclusion

In this paper, we offer a multidimensional scheme to measure the ethics of political PR specialists, since one-dimensional frameworks used in previous research did not account for the contextual variety of human moral reasoning or apply it to the investigation of the moral reasoning of the members of the Russian political PR industry. In particular, we examine the ethical principles of 16 leading “players” in the market.

Our first finding was contrary to the research of [77]. They showed that for Russian students who study PR, ethical consequences of their professional activity is less important than for the students from other countries or the negative social perceptions and media portrayals of people who work in the political PR industry [78, 79]. Instead, our findings are in line with [5, 80], who revealed that Russian political PR professionals are bothered by ethical outcomes of their professional actions. However, as in the studies of the ethical principles of PR specialist in Russia, their research was conducted mainly on student populations. Nevertheless, we suppose that additional research is needed to reveal whether the ethical principles of PR professional in Russia and in the other countries depend on the level of education (student / working professional) or on the branch of the industry.

Second, regarding the nature of moral reasoning, we show that the ethics of political PR professionals depend on the perception of whom their actions could affect. These include clients, other professionals, their professional organizations, the public, governmental structures, and other politicians. Moreover, we discovered all five moral foundations from the range suggested by Haidt in the narratives of our informants. However, three foundations, “fairness/reciprocity,” “in-group/loyalty” and “purity/sanctity,” were referred to more than the other two (“care/harm” and “authority/respect”). Such results are in line with previous papers that show that Russian PR specialists are less likely than US ones to think about how their actions affect the public good [81]. We suppose that Russian political PR professionals spoke less about the potential outcomes of their actions regarding the foundations because they value relations with the members of their professional community more than what is thought by clients or people who represent public opinion. At the same time, our study features only market leaders. Since the political PR industry in Russia is shown to be fragmented, we suppose that the foundations of less powerful members of the industry could be of different character [5].

Third, like [5, 77, 82], we show that the attitudes toward certain actors (the public, clients, their organizations, other PR specialists) depend on the normative legal structures that are present in the country, ethical codes in the industry, and informal collective ethical principles established in the Russian political PR industry. Our informants claimed that the moral principles that they used are inherited from parents and have a universal character. Moreover, for them, general human values are more important than formal codes. This also is in line with the research of [4]. Future research aimed at enriching the knowledge of the multifaceted normative context that influences the professional ethics of the political PR professionals could consider analyzing situations in which the normative structures contradict each other. Such a study could reveal the hierarchy of the normative systems that are developed in the community of PR professionals. Such a hierarchy may be used to improve the official ethical codes or create new ones.

Finally, we found that attitudes toward the subjects to whom political PR specialists apply ethical principles depend on each other. For instance, being polite to other professionals could also presuppose being brutal to the political figures that these other professionals represent. This observation leads us to the idea of the networked structure of ethical orientations that the political PR professionals may have developed in their professional practice. Furthermore, our research was not targeted on the analysis of moral agency prescription by these professionals, but this topic should be investigated further because understanding whom PR professionals consider moral agents could explain why some moral foundations are more involved in their moral reasoning than others. For example, if these professionals do not think that the public is a collective agent [83] that could be harmed and be under threat because of their actions, they reasonably will not take the security of the public into consideration while making their professional decisions.

## Limitations

Our study is not without limitations. First, the sample includes mainly male political PR professionals. This reflects the male-domination of the Russian political PR industry [84]. Moreover, our choice of informants was explained by this demographic-specific field. Therefore, we suppose that future studies could analyze differences in moral reasoning of political PR specialists based on gender. It could be that the moral foundations of female political PR professionals are significantly different from the male ones. For instance, they could reflect gender ideology and culturally specific role expectations [85]. Second, we interviewed the leaders of the Russian political PR industry, and their perceptions of professional ethics could be different from those of outsiders or simply from the less privileged Russian regions [4], where these people usually do not work. Therefore, additional studies are required to understand whether there is a division in moral values in political PR specialists who come from parts of Russia with various levels of economic development. Third, only specialists from Russia took part in this research, and the moral foundations of specialists from the other countries could be different because of institutional and other aspects related to the functioning of the PR industry in those countries [49]. Fourth, we employed a qualitative approach to our elicitation of the participants’ moral foundations. In prospective studies, a similar method could measure the prevalence of foundations among professionals and in their documents (their codes of ethics and other formal and informal narratives).
